# Identification of active sequences in HIV-1 molecular transmission networks using machine learning: a cross-sectional study in Hainan Province, south China

**DOI:** 10.3389/fcimb.2026.1925404

**Published:** 2026-10-01

**Authors:** Dee Yu, Nengde Zhang, Xin Chen, Ruolan Fu, Zhenjiao Zhang, Chunyun Lu, Jing Su, Qionggui Zhou, Qiongjun Xu, Haiqing Xu, Bo Bi, Zhaoqian Wang, JindongDing Petersen

**Affiliations:** 1 Key Laboratory of Tropical Translational Medicine of Ministry of Education, School of Public Health, Hainan Academy of Medical Sciences, Hainan Medical University, Haikou, Hainan, China; 2 University of the West of England (UWE) College, School of International Education, Hainan Medical University, Haikou, Hainan, China; 3 Department of AIDS Prevention and Control, Hainan Provincial Center for Disease Control and Prevention, Haikou, Hainan, China

**Keywords:** active sequence, cross-sectional study, gradient boosting model, HIV-1, molecular transmission network

## Abstract

**Background:**

New HIV-1 infections continue to increase in Hainan Province, a major tourist destination in southern China characterized by high population mobility and diverse viral subtypes. Understanding transmission dynamics within molecular networks is critical for precision prevention.

**Methods:**

We conducted a cross-sectional study of participants living with HIV-1 in Hainan Province between 2021 and 2025. HIV-1 molecular transmission networks were constructed using HIV-TRACE. Sequences from individuals diagnosed by the end of 2023 were used to construct the baseline network, and sequences from newly reported cases during 2024–2025 were added to form the final network. Active sequences were defined as those from newly reported cases in 2024–2025 or those directly linked to newly reported cases within the final network. Multivariable logistic regression was used to identify factors independently associated with active sequences, and gradient boosting to assess variable importance.

**Results:**

Among 2,342 sequences, 1,071 (45.7%) were included in the final molecular transmission network, comprising 505 (47.2%) active sequences. Multivariable analysis showed that male sex (*aOR* = 2.340, 95% CI:1.416–3.866), age ≥50 years (*aOR* = 3.806, 95% CI:2.485–5.830), drug resistance (*aOR* = 1.777, 95% CI:1.227-2.575), and other subtypes (*aOR* = 2.204, 95% CI:1.560-3.115) were independently associated with active sequences. The gradient boosting model corroborated these findings, ranking age at diagnosis, HIV-1 subtype, drug resistance, and sex as the top four factors discriminating active sequences.

**Conclusions:**

Our findings indicate that older age (≥50 years), less prevalent subtypes, drug resistance, and male sex are key factors associated with active transmission in Hainan. Targeted interventions, encompassing routine screening for older adults, baseline resistance testing, and enhanced molecular surveillance, should be prioritized to curb ongoing HIV-1 dissemination.

## Introduction

HIV remains a major challenge for global public health. By the end of 2024, approximately 40.8 million people were living with HIV globally, and 1.3 million new infections occurred that year (UNAIDS[EB], 2025). In the same year, 101,600 new HIV/AIDS cases were reported in China (National Center for AIDS/STD Control and Prevention, 2025a). Within China, the transmission landscape have shifted from injection drug use and blood-borne pathways toward more clandestine routes, with sexual contact now the predominant route (National Center for AIDS/STD Control and Prevention, 2025b). Conventional field epidemiology struggles to trace HIV transmission chains and identify high-risk populations, owing to difficulties in obtaining private information and the limited reliability of the data (He, 2021). Therefore, novel technological approaches are urgently needed to enhance HIV prevention and control.

HIV molecular surveillance has been implemented in China for many years, yielding a large repository of HIV genetic sequences. From these sequences, HIV-1 molecular transmission networks can be constructed on the basis of genetic distance, providing a novel tool for HIV prevention and control (Wertheim et al., 2018). In such a network, each node represents an HIV/AIDS case (Zhang et al., 2020), and an edge between two nodes indicates that the genetic distance between their viral sequences falls below a predefined threshold, suggesting a potential transmission link. Networks built in this way enable the rapid identification of patients infected with genetically similar viral strains. When integrated with epidemiological and behavioral information, they can further reveal active transmission clusters, super-spreaders, and geographic transmission hotspots (Xu et al., 2024; Li et al., 2026).

Most previous studies of HIV-1 molecular transmission networks have focused on static snapshots at single time points. Yet HIV infections persist over time, and the networks they form are inherently dynamic (Chan et al., 2015), shaped by epidemic trends, population mobility, and socioeconomic conditions (Eshraghian et al., 2020). Static structures alone cannot fully explain transmission dynamics or potential spread risks. Research on active HIV transmission also remains limited, with one study (Oster et al., 2018) defining a molecular cluster as active when it contained at least five newly diagnosed cases within the preceding 12 months. Although designed to prioritize individuals for intervention, this cluster-level screening may inadvertently include nodes that have been inactive for long periods. This issue is particularly significant for slowly evolving HIV strains such as CRF07_BC, which tend to form large clusters of hundreds of nodes under loose genetic distance thresholds. Consequently, cluster-level screening can generate substantial false positives, straining already limited public health resources.

To address this limitation, our study retains the fundamental epidemiological rationale of the cluster-level approach, anchoring the active transmission to newly reported cases, but refines the identification unit from the entire cluster to individual nodes. Rather than requiring an entire cluster to meet the activity criteria, we label only those nodes with direct genetic links to newly reported cases. This node-level refinement aims to enhance specificity, improve computational transparency, and provide a reproducible complementary strategy to existing cluster-based methods. It is particularly suited for routine surveillance settings where resources are limited.

Hainan, China’s southernmost province, is an island that borders the provinces of Guangdong and Guangxi to the north, and lying close to Vietnam, Laos, Indonesia, and the Philippines. By the end of 2022, 4,286 people were living with HIV (PLWH) in the Province, with cases concentrated mainly in Haikou and Sanya. HIV-1 strains circulating in Hainan are highly diverse (Yu et al., 2023), and local transmission patterns have grown increasingly complex. Against this background, we conducted a cross-sectional study to construct a dynamic HIV-1 molecular transmission network, identify active sequences within the network, and apply machine learning algorithms to determine the key factors associated with these active sequences. The findings should help guide precision intervention and the efficient allocation of public health resources.

## Methods

### Study population

We enrolled PLWH in Hainan Province between 2021 and 2025. Participants were eligible if they were aged 18 years or older and had provided written informed consent. Those who refused to participate in the survey or blood collection, or who had severe mental disorders, were excluded.

### Study design and data collection

We adopted a cross-sectional design. The HIV *pol* sequences were obtained from the Hainan HIV molecular surveillance project, launched in January 2021. Participants were recruited in two phases from two sources. First, individuals with confirmed HIV infection receiving antiretroviral therapy (ART) were recruited from HIV treatment centers across the cities and counties of Hainan Province. Second, newly diagnosed HIV/AIDS cases were recruited from local centers for disease control and prevention. From each participant, 5 mL of venous blood was collected for laboratory testing.

Baseline information of the participants was obtained from the China Information System for Disease Control and Prevention, covering (1) basic demographic information (age, sex, marital status, ethnicity, education, household registration location, current residence, and occupation); (2) testing history for HIV and other sexually transmitted infections; (3) high-risk behavioral history (transmission route and number of sexual partners); (4) CD4^+^ T-cell count; and (5) other variables, such as a history of trimethoprim-sulfamethoxazole (TMP-SMX) prophylaxis.

### HIV-1 cDNA/RNA extraction and sequencing

For participants receiving ART, DNA was extracted from 200 μL of whole blood using the Nucleic Acid Extraction or Purification Reagent (catalog No. 10T146; Xi’an Tianlong Science and Technology Co., Ltd., Xi’an, China) on the Tianlong NP968 automated extraction system, following the manufacturer’s protocol. A 1,356-bp fragment of the HIV-1 *pol* gene (nucleotides 2086–3441 of the reference strain HXB2) was then amplified by polymerase chain reaction (PCR) for sequencing. For ART-naïve participants, HIV-1 RNA was extracted from plasma using the Viral DNA/RNA Extraction Kit (CDC) (catalog No. 10T014; Xi’an Tianlong Science and Technology Co., Ltd., Xi’an, China) on the same automated system. For reverse transcription (RT-PCR), 2 µL of the RNA eluate was used as the template in a total reaction volume of 25 µL, with the PrimeScript One Step RT-PCR Kit Ver.2 (TaKaRa Bio Inc., Dalian, China), which provides reverse transcriptase and reaction buffer in a premixed master mix.

Detailed information on primer sequences, thermal cycling parameters, and reaction conditions for HIV-1 *pol* amplification is summarized in **Supplementary Tables S1–S3**. The positive RT-PCR products underwent Sanger sequencing. Only sequences with ≤5% mixed bases and a length of ≥1000 bp were retained. In total, 2,342 *pol* sequences were obtained from 3,729 participants.

### Drug resistance analysis

All HIV-1 sequences were uploaded to the Stanford HIV Drug Resistance Database (https://hivdb.stanford.edu/hivdb/by-sequences/) for drug resistance analysis. Resistance to 22 antiretroviral drugs was assessed, spanning non-nucleoside reverse transcriptase inhibitors, nucleoside reverse transcriptase inhibitors, and protease inhibitors. For each drug, the resistance level was inferred from the Stanford mutation scores and categorized as sensitive, potentially resistant, low-level resistant, moderately resistant, and highly resistant. Participants showing low-level resistance or higher to any drug were defined as having drug resistance.

### HIV-1 molecular transmission network analysis

Pairwise genetic distances between sequences were calculated with HIV-TRACE under the Tamura-Nei 93 model. Molecular transmission networks were constructed across genetic distance thresholds from 0.001 to 0.020, in steps of 0.001, and the number of clusters and of nodes included at each threshold were compared to identify the optimal value, favoring a relatively high number of both.

Overall, 2,342 HIV-1 sequences from Hainan Province were used for network construction. The number of clusters peaked at a genetic distance threshold of 0.009, which was therefore selected for constructing the molecular transmission network (**Figure 1**).

**Figure 1:**
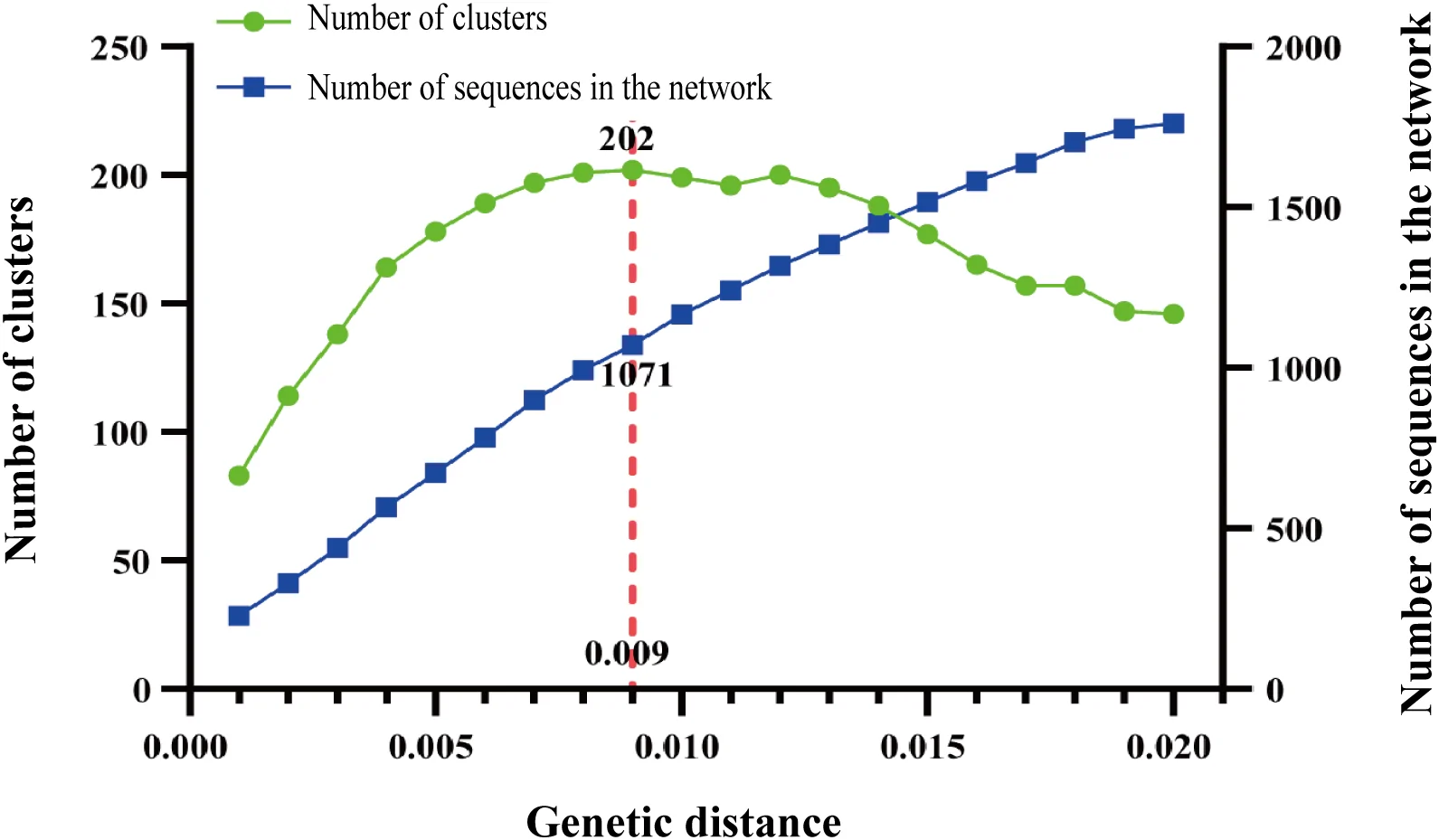
Selection of the optimal genetic distance threshold for network construction. The numbers of clusters and sequences included in the network are shown for each genetic distance threshold from 0.001 to 0.020. The threshold of 0.009 was selected as optimal because it yielded the highest number of clusters while retaining a relatively high number of included sequences.

Sequences from individuals diagnosed by the end of 2023 formed the baseline network, to which sequences from newly reported cases during 2024–2025 were added at the same threshold. The final molecular network was annotated and visualized in Cytoscape v3.6.1.

### Outcomes

We adopted a node-level strategy to define the outcome variable. Epidemiologically, a node directly linked to a newly reported case represents an individual separated from an ongoing transmission event by a single genetic link. Specifically, a sequence was labeled as active when it either (1) originated from a newly reported case during 2024–2025 and was included in the final molecular transmission network, or (2) originated from an individual diagnosed by the end of 2023 but was linked to a newly reported 2024–2025 sequence within the final network. All remaining sequences were labeled as inactive. This definition has been previously applied in two independent Chinese HIV-1 molecular network studies (Li, 2021; Cheng, 2024), supporting its epidemiological plausibility.

Since ongoing transmission naturally spans calendar years, cases newly reported in 2024–2025 may still be linked to cases from earlier periods. Our labeling strategy follows the CDC-based active cluster framework, and aims to capture the current leading edge of the network rather than to establish temporal independence between cohorts.

### Definitions

Employment status: Participants employed as teachers, commercial and service personnel, cadres, company employees, and physicians were classified as having stable employment, while those recorded as housework/unemployed, farmers, self-employed or freelance workers, students, or others were classified as lacking stable employment.

Migrant population: Participants whose current residence differed from their household registration location were defined as migrants.

Economic regional classification: According to the economic classification criteria of the Hainan Statistical Yearbook 2024, the cities and counties of Hainan Province were grouped into three tiers, namely Category I (Haikou, Danzhou including Yangpu, Sanya, and Chengmai); Category II (Wenchang, Qionghai, Wanning, Dongfang, Lingshui, Lingao, and Ledong); and Category III (Changjiang, Ding’an, Tunchang, Baoting, Qiongzhong, Baisha, and Wuzhishan).

### Statistical analysis

Statistical analyses were conducted in R v.4.5.0. Categorical variables were summarized as frequencies and percentages and compared between individuals included in the network and those not included, using chi-square tests. Univariable logistic regression was employed to identify factors associated with active sequences, and variables with *P* < 0.05 in the univariable analysis were entered into the multivariable logistic regression model.

### Gradient boosting model

The gradient boosting model was fitted to the 1,071 sequences included in the molecular transmission network, of which 505 (47.2%) were active. Six categorical predictors were incorporated, namely sex, age at diagnosis, education level, transmission route, drug resistance, and HIV-1 subtype.

Gradient boosting was implemented with XGBoost (eXtreme Gradient Boosting). Hyperparameters were tuned by grid search over the following ranges, namely learning rate (eta, {0.03, 0.10}), maximum tree depth (max_depth, {2, 3}), subsample ratio (subsample, {0.6, 0.8, 1.0}), column subsampling ratio (colsample_bytree, {0.6, 0.8, 1.0}), and the number of boosting rounds (n_estimators, {200, 500}), yielding 32 candidate combinations. Model selection used stratified 10-fold cross-validation repeated five times, with the mean area under the receiver operating characteristic curve (AUC) as the selection criterion. The optimal configuration was n_estimators = 200, max_depth = 2, eta = 0.03, colsample_bytree = 1.0, min_child_weight = 5, and subsample = 0.8.

SHAP (SHapley Additive exPlanations) values were computed in an out-of-fold manner. In each of the 10 outer folds, the model was refit on the remaining folds, and exact TreeSHAP contributions were calculated for the held-out sequences, ensuring that each sequence received out-of-sample attributions. Contributions were expressed on the log-odds scale and were additive, such that for any given sequence, the sum of the contribution values plus the model intercept equaled its linear predictor. Additivity was verified numerically within each fold. Since all predictors were categorical and had been expanded into indicator columns, SHAP values were aggregated back to the original variable level by summing, for each sequence, the signed contributions of all indicator columns belonging to the same variable. Variable importance was then defined as the mean absolute per-sequence contribution of each variable.

## Results

### Demographic characteristics of participants

Overall, 2,342 *pol* sequences were analyzed, with 79.8% from cases diagnosed by the end of 2023 and 20.2% from cases newly reported during 2024–2025. Of these, 89.5% were male, with no difference in sex distribution between the two periods. Notable temporal shifts emerged, with participants diagnosed in 2024–2025 being significantly older (35.0% vs. 15.7% aged ≥50 years), less likely to have a college degree or higher (33.5% vs. 42.1%), and more likely to be married or in a partnership (33.4% vs. 25.3%). Regarding transmission routes, homosexual contact persisted as the predominant route overall, accounting for 52.6%. However, the proportion declined from 54.7% to 44.3% in 2024–2025, while heterosexual contact increased from 40.8% to 51.5%. In terms of HIV-1 subtypes, CRF01_AE (40.7%) and CRF07_BC (35.1%) were the predominant subtypes overall. Geographically, the proportion of participants from Category I regions decreased from 77.0% to 67.3%, accompanied by proportional increases in Category II and III regions. Collectively, these findings indicate a shifting epidemiological profile characterized by an older age demographic, lower socioeconomic status, and an increase in heterosexual transmission in recent years (**Table 1**).

**Table 1:** Demographic characteristics of participants by diagnosis period, n (%).

Variables	Total	By the end of 2023	2024–2025
Total	2342 (100.0)	1868 (79.8)	474 (20.2)
Sex			
Male	2097 (89.5)	1673 (89.6)	424 (89.5)
Female	245 (10.5)	195 (10.4)	50 (10.5)
Age at diagnosis (years)			
<20	110 (4.7)	89 (4.8)	21 (4.5)
20~29	839 (35.8)	730 (39.1)	109 (23.0)
30~39	605 (25.8)	494 (26.4)	111 (23.4)
40~49	328 (14.0)	261 (14.0)	67 (14.1)
≥50	460 (19.7)	294 (15.7)	166 (35.0)
Education level			
Primary school or below	290 (12.4)	197 (10.5)	93 (19.6)
Junior high school	579 (24.7)	453 (24.3)	126 (26.6)
Senior high school/technical secondary school	527 (22.5)	431 (23.1)	96 (20.3)
College degree or above	946 (40.4)	787 (42.1)	159 (33.5)
Marital status			
Married or with partner	636 (27.2)	473 (25.3)	163 (33.4)
Unmarried	1437 (61.4)	1190 (63.7)	247 (52.1)
Divorced or widowed	269 (11.4)	205 (11.0)	64 (13.5)
Ethnicity			
Han	2048 (87.5)	1655 (88.6)	393 (82.9)
Li	218 (9.3)	153 (8.2)	65 (13.7)
Others	76 (3.2)	60 (3.2)	16 (3.4)
Transmission route			
Homosexual	1232 (52.6)	1022 (54.7)	210 (44.3)
Heterosexual	1005 (42.9)	761 (40.8)	244 (51.5)
Others	105 (4.5)	85 (4.5)	20 (4.2)
Subtype			
CRF01_AE	954 (40.7)	765 (41.0)	189 (39.9)
CRF07_BC	823 (35.1)	669 (35.8)	154 (32.5)
Others	565 (24.1)	434 (23.2)	131 (27.6)
Economic regional classification			
Category I	1758 (75.1)	1439 (77.0)	319 (67.3)
Category II	378 (16.1)	282 (15.1)	96 (20.3)
Category III	206 (8.8)	147 (7.9)	59 (12.4)

### Identification of HIV-1 active sequences

A total of 1,071 sequences were included in the final molecular transmission network, giving a clustering rate of 45.7% (1,071/2,342). Of these, 566 (52.9%) were inactive sequences and 505 (47.2%) were active sequences (**Figure 2**).

**Figure 2:**
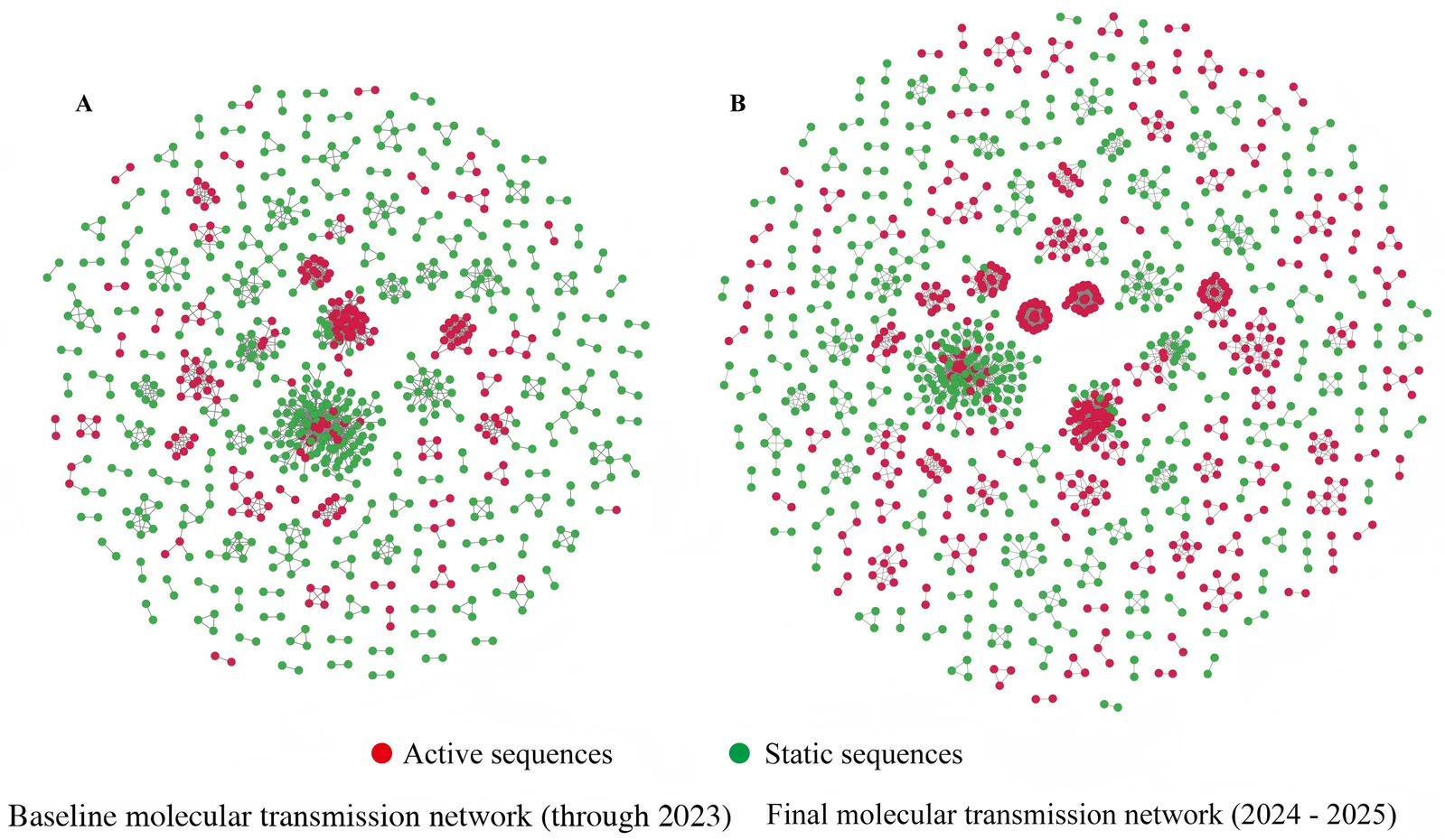
HIV-1 molecular transmission network in Hainan Province. **(A)** Baseline molecular transmission network (sequences diagnosed by the end of 2023); **(B)** Final molecular transmission network (baseline plus sequences newly reported during 2024–2025). Nodes represent individual HIV-1 sequences, with colors indicating sequence status (red for active sequences and blue for inactive sequences). Edges represent genetic links between sequences at the pairwise genetic distance threshold of 0.009.

**Table 2** compares the characteristics of individuals included in the molecular network with those not included. Significant differences were observed in sex, age at diagnosis, and HIV-1 subtype (*P* < 0.05), but not in education level, marital status, ethnicity, transmission route, or economic status (*P*>0.05).

**Table 2:** Characteristics of individuals included in the molecular network and those not included, n (%).

Variables	Total	Not included in the network	In the network	*χ²*	*P*
Total	2342 (100.0)	1271 (54.3)	1071 (45.7)		
Sex				4.725	0.030
Male	2097 (89.5)	1122 (53.5)	975 (46.5)		
Female	245 (10.5)	149 (60.8)	96 (39.2)		
Age at diagnosis (years)				32.077	<0.001
<20	110 (4.7)	62 (56.4)	48 (43.6)		
20~29	839 (35.8)	471 (56.1)	368 (43.9)		
30~39	605 (25.8)	352 (58.2)	253 (41.8)		
40~49	328 (14.0)	190 (57.9)	138 (42.1)		
≥50	460 (19.7)	196 (42.6)	264 (57.4)		
Education level				5.865	0.118
Primary school or below	290 (12.4)	139 (47.9)	151 (52.1)		
Junior high school	579 (24.7)	313 (54.1)	266 (45.9)		
Senior high school or technical secondary school	527 (22.5)	290 (55.0)	237 (45.0)		
College degree or above	946 (40.4)	529 (55.9)	417 (44.1)		
Marital status				1.556	0.459
Married or with partner	636 (27.2)	333 (52.4)	303 (47.6)		
Unmarried	1437 (61.4)	794 (55.3)	643 (44.7)		
Divorced or widowed	269 (11.4)	144 (53.5)	125 (46.5)		
Ethnicity				3.302	0.192
Han	2048 (87.5)	1105 (54.0)	943 (46.0)		
Li	218 (9.3)	117 (53.7)	101 (46.3)		
Others	76 (3.2)	49 (64.5)	27 (35.5)		
Transmission route				8.010	0.018
Homosexual	1232 (52.6)	656 (53.2)	576 (46.8)		
Heterosexual	1005 (42.9)	553 (55.0)	452 (45.0)		
Others	105 (4.5)	62 (59.0)	43 (41.0)		
Subtype				36.029	<0.001
CRF01_AE	954 (40.7)	541 (56.7)	413 (43.3)		
CRF07_BC	823 (35.1)	441 (53.6)	382 (46.4)		
Others	565 (51.2)	289 (59.6)	276 (48.8)		
Economic classification				2.553	0.279
Category I	1758 (75.1)	966 (54.9)	792 (45.1)		
Category II	378 (16.1)	191 (50.5)	187 (49.5)		
Category III	206 (8.8)	114 (55.3)	92 (44.7)		

### Characteristics of HIV-1 sequences in the network and of active sequences

Among the individuals whose sequences were included in the network, 91.0% were male, 60.0% were unmarried, and 39.0% had a college degree or higher. Homosexual contact was the primary transmission route (53.8%). CRF01_AE (38.6%) and CRF07_BC (35.7%) were the predominant subtypes, together accounting for nearly three-quarters of all sequences. (**Table 3**).

**Table 3:** Characteristics of HIV-1 active sequences and univariable analysis of factors associated with active sequences.

Variables	Sequences in the network, n (%)	Active sequences, n (%)	aOR (95% CI)
Total	1071 (100.0)	505 (47.2)	
Sex			
Male	975 (91.0)	470 (48.2)	1.62 (1.05-2.50)
Female	96 (9.0)	35 (36.5)	1
Age at diagnosis (years)			
<20	48 (4.5)	26 (54.2)	1.75 (0.96-3.21)
20~29	368 (34.4)	148 (40.2)	1
30~39	253 (23.6)	110 (43.5)	1.14 (0.82-1.58)
40~49	138 (12.9)	54 (39.1)	0.95 (0.64-1.42)
≥50	264 (24.6)	167 (63.3)	2.55 (1.84-3.54)
Education			
Primary school or below	151 (14.1)	86 (57.0)	1
Junior high school	266 (24.8)	119 (44.7)	0.61 (0.40-0.91)
Senior high school	237 (22.1)	111 (46.8)	0.66 (0.44-1.00)
College degree or above	417 (39.0)	189 (45.3)	0.62 (0.43-0.91)
Marital status			
Married or with partner	303 (28.3)	147 (48.5)	1
Unmarried	643 (60.0)	290 (45.1)	0.87 (0.66-1.14)
Divorced or widowed	125 (11.7)	68 (54.4)	1.26 (0.83-1.92)
Ethnicity			
Han	943 (88.0)	440 (46.7)	1
Li	101 (9.5)	50 (49.5)	1.12 (0.74-1.69)
Others	27 (2.5)	15 (55.6)	1.42 (0.66-3.08)
History of sexually transmitted infections			
Yes	206 (19.2)	103 (50.0)	1
No	771 (72.0)	347 (45.0)	0.81 (0.60-1.11)
Unknown	94 (8.8)	55 (58.5)	1.41 (0.86-2.31)
Transmission route			
Homosexual	576 (53.8)	267 (46.4)	1
Heterosexual	452 (42.2)	226 (50.0)	1.21 (0.94-1.57)
Others	43 (4.0)	12 (27.9)	0.50 (0.25-1.00)
Having a stable job			
Yes	379 (35.4)	173 (45.6)	1
No	692 (64.6)	332 (48.0)	1.09 (0.85-1.41)
Baseline CD4^+^ T-cell count (cells/μL)			
<200	336 (31.4)	166 (49.4)	1
200~349	402 (37.5)	191 (47.5)	0.92 (0.69-1.24)
≥350	333 (31.1)	148 (44.4)	0.81 (0.60-1.11)
Drug resistance			
No	897 (83.8)	398 (44.4)	1
Yes	174 (16.2)	107 (61.5)	2.00 (1.44-2.79)
Number of sexual partners			
<3	474 (44.2)	226 (47.7)	1
3~10	353 (33.0)	157 (44.5)	0.88 (0.66-1.15)
>10	244 (22.8)	122 (50.0)	1.09 (0.80-1.49)
Migrant population			
Yes	632 (59.0)	298 (47.2)	1
No	439 (41.0)	207 (47.2)	1.00 (0.78-1.27)
History of TMP-SMX prophylaxis			
Yes	193 (18.0)	97 (50.3)	1
No	836 (78.1)	380 (45.5)	0.82 (0.60-1.13)
Unknown	42 (3.9)	28 (66.7)	1.98 (0.98-3.99)
Subtype			
CRF01_AE	413 (38.6)	174 (42.1)	1
CRF07_BC	382 (35.7)	158 (41.4)	0.97 (0.74-1.32)
Others	276 (25.7)	173 (62.7)	2.32 (1.69-3.20)
Economic regional classification			
Category I	792 (73.9)	359 (45.3)	1
Category II	187 (17.5)	96 (51.3)	1.27 (0.93-1.75)
Category III	92 (8.6)	50 (54.3)	1.44 (0.93-2.22)

TMP-SMX, trimethoprim-sulfamethoxazole.

Within the final molecular transmission network, 63.3% of the sequences from participants aged ≥50 years were active. Other subtypes (non-CRF01_AE/non-CRF07_BC) showed notably high proportions of active sequences (62.7%). Of the sequences from participants with drug resistance, 61.5% were active. (**Table 3**).

### Factors associated with active sequences

Multivariable logistic regression showed that male sex (*aOR* = 2.340, 95% CI: 1.416–3.866), age ≥50 years (*aOR* = 3.806, 95% CI: 2.485–5.830), drug resistance (*aOR* = 1.777, 95% CI: 1.227–2.575), and other subtypes (*aOR* = 2.204, 95% CI: 1.560–3.115) were independently associated with increased odds of being an active sequence. However, given the inherent sex imbalance in the study population (89.5% male), the association with male sex should be interpreted with caution and may be specific to the epidemiological context of Hainan Province (**Figure 3**).

**Figure 3:**
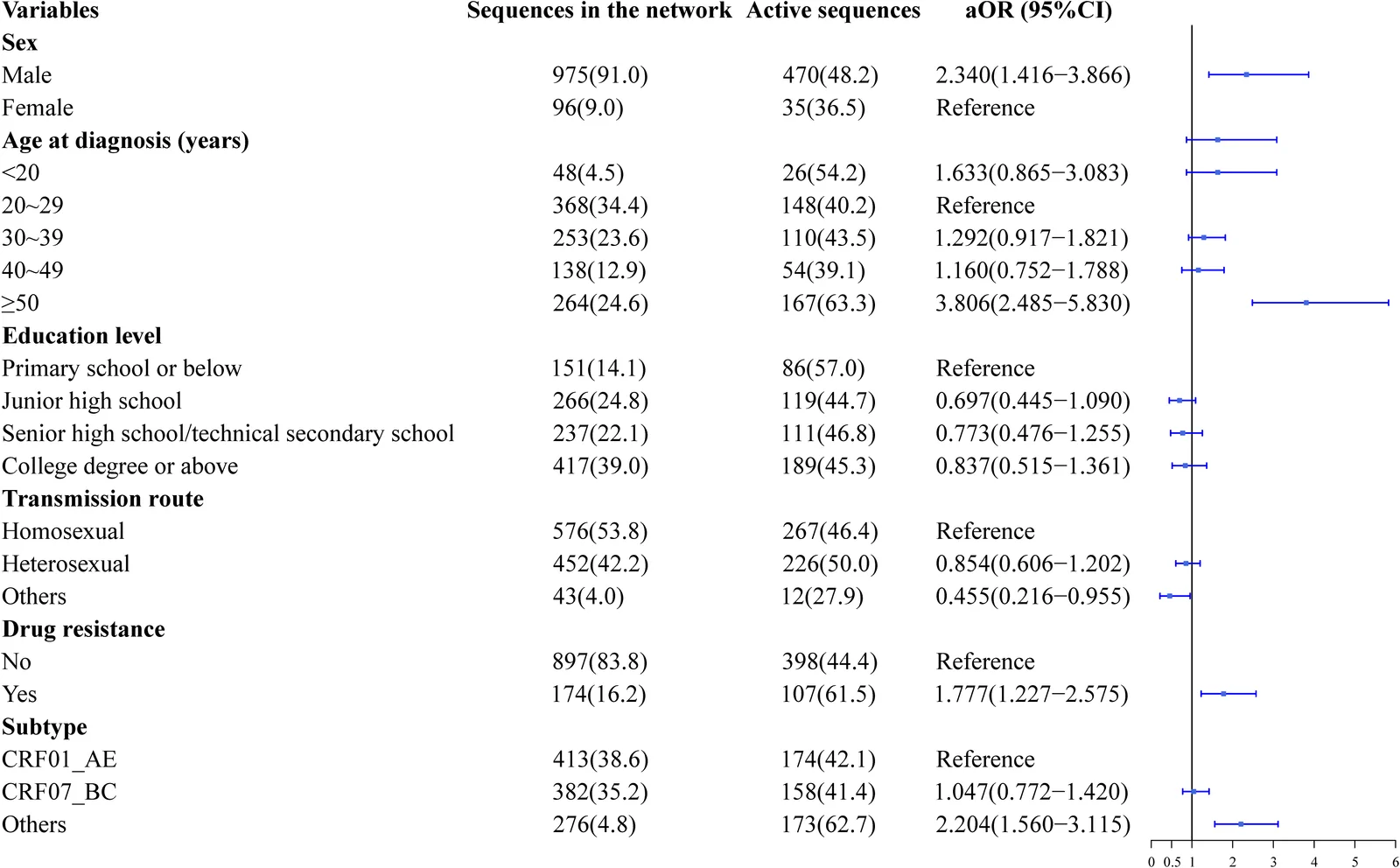
Forest plot of multivariable logistic regression analysis of factors associated with active sequences. All estimates were adjusted for sex, age at diagnosis, education level, transmission route, drug resistance, and HIV-1 subtype. A vertical dashed line at *aOR* = 1 indicates the null value. Error bars represent 95% CIs; associations were considered statistically significant when the 95% CI did not cross 1. *aOR*, adjusted odds ratio; CI, confidence interval.

### Model performance and variable importance from gradient boosting

The gradient boosting model achieved an out-of-fold AUC of 0.660 (95% CI: 0.628–0.693) (**Figure 4B**), with good calibration (calibration intercept=−0.011, calibration slope=0.949, Brier score=0.229) (**Figure 4C**). Age at diagnosis contributed most to model prediction (mean |SHAP| = 0.375, accounting for 36.4% of the total importance), followed by HIV-1 subtype (0.277, 26.9%), drug resistance (0.144, 14.0%), and sex (0.113, 10.9%). Education level (0.100, 9.4%) and transmission route (0.042, 3.9%) ranked lowest (**Figure 4A**).

**Figure 4:**
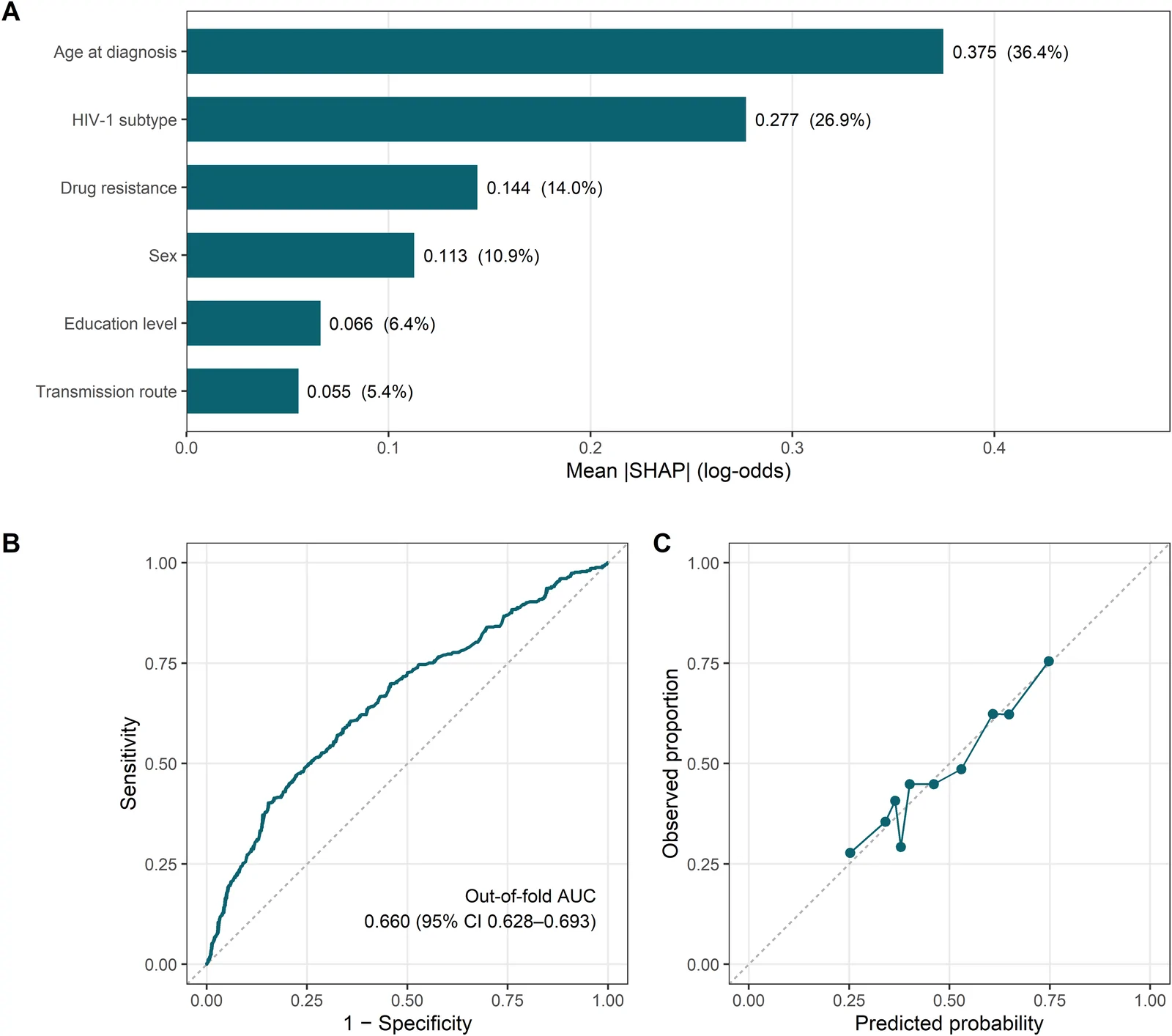
Gradient boosting machine (XGBoost) model performance and SHAP-based variable importance for identifying active sequences. **(A)** Variable importance ranking quantified by mean absolute SHAP values (log-odds scale), with percentages indicating the relative contribution of each factor to the total importance. **(B)** Receiver operating characteristic (ROC) curve showing the out-of-fold performance of the gradient boosting model, with an area under the curve (AUC) of 0.660 (95% CI: 0.628–0.693). The diagonal dashed line indicates no discriminative ability (AUC = 0.5). **(C)** Calibration plot comparing predicted probabilities (x-axis) against observed proportions (y-axis). The dashed diagonal line indicates perfect calibration. SHAP, SHapley Additive exPlanations; CI, confidence interval.

## Discussion

In this study, we combined a cross-sectional HIV-1 molecular transmission network approach with logistic regression and gradient boosting to identify factors associated with active sequences. Of the 2,342 sequences obtained from people living with HIV-1 in Hainan Province, 45.7% were included in the network, and 47.2% of these were classified as active, indicating substantial ongoing transmission. Multivariable logistic regression identified sex, age at diagnosis, drug resistance, and HIV-1 subtype as independent factors, a pattern corroborated by the gradient boosting model, which ranked these as the top discriminative features. Given Hainan’s high population mobility, these findings inform targeted interventions to limit cross-regional HIV-1 dissemination.

### Age ≥ 50 years as a key demographic risk factor

The proportion of active sequences was significantly higher among participants aged ≥50 years (63.3%) than among younger participants. Age was further confirmed as an independent factor in multivariable analysis (*aOR* = 2.340) and was ranked as the most important feature in the machine learning model. This finding is particularly concerning for Hainan Province, where HIV infections among older adults have been increasing in recent years, predominantly through heterosexual transmission, a pattern consistent with the national figure of 91.24% for heterosexual transmission in this age group (Hou et al., 2024).

Behavioral and social factors, rather than biological susceptibility alone, likely underlie this pattern. In Hainan, a substantial proportion of older cases (≥50 years) have been attributed to unprotected commercial sex, frequently coupled with limited awareness of HIV prevention, particularly in rural regions (Lin et al., 2018). Condom use in this age group is often inconsistent, and HIV testing may be delayed due to misconception, leading to higher rates of virologic failure (Shan et al., 2026). Additionally, age-related immunological changes and comorbidities may compromise treatment responses. Together, these factors may facilitate sustained transmission within relatively stable and interconnected sexual networks, resulting in the higher proportions of active sequences observed among older individuals. Similar age-related clustering patterns have been reported in molecular epidemiological studies conducted in other regions of China, supporting the plausibility of these mechanisms (Zhang et al., 2023). Age-specific interventions are therefore warranted, particularly in rural regions.

### Male predominance in ongoing HIV-1 transmission networks

Male sex emerged as a strong independent factor associated with active sequences, with males having 2.34-fold higher odds than females. Among the individuals included in the network, 91.0% were male, and 48.2% of these had active sequences. This male predominance is consistent with China’s HIV epidemiology, where most adults living with HIV are male (Chen et al., 2024). Unlike in other regions, transmission among men who have sex with men (MSM) predominates in Hainan Province. MSM typically have higher partner turnover and greater network connectivity, facilitating rapid viral dissemination within sexual networks (Yang et al., 2024). Furthermore, behavioral factors, such as inconsistent condom use, may contribute to prolonged undiagnosed intervals, thereby expanding opportunities for onward transmission. In Hainan, where tourism and migrant labor attract large male populations, targeted outreach to sexually active males, including migrant workers, should be prioritized to disrupt ongoing transmission chains.

### Subtype-specific transmission dynamics

HIV-1 subtype also emerged as an independent factor associated with active sequences and ranked as the second most important predictor in the machine learning model. The markedly higher proportions of active sequences among certain subtypes suggest that these subtypes may possess a transmission advantage or circulate within more dynamic transmission networks.

CRF01_AE was the predominant circulating subtype in Hainan Province in the early stage of the local epidemic (Deng et al., 2009). CRF07_BC subsequently emerged and gradually became the second most prevalent subtype in the region. Currently, these two subtypes remain predominant in Hainan, followed by CRF55_01B, CRF65_cpx, and CRF08_BC (Zhang et al., 2026). Compared with CRF01_AE and CRF07_BC, these three subtypes are younger circulating recombinant forms in the local epidemic. Our previous study found that CRF65_cpx was introduced into Hainan through the MSM community in 2013, and the virus then spread within this population, giving rise to a large and stable transmission cluster (Yu et al., 2024). In addition, CRF65_cpx showed a high clustering rate (Yu et al., 2024, Yu et al., 2025). Therefore, CRF65_cpx sequences are more likely to be classified as active, which may reflect the higher transmissibility of this subtype and its centrality within the molecular transmission network.

CRF08_BC showed marked geographic clustering in specific areas of Hainan Province and was transmitted primarily through heterosexual contact (Zhang et al., 2026). Such demographic and geographic concentration likely facilitates denser transmission links within the molecular network, increasing the likelihood that sequences become active. Comparable geographic clustering of CRF08_BC has been documented in Zhejiang Province (Fan et al., 2025; Zhao et al., 2025). Close monitoring of these subtypes, together with timely targeted interventions, is therefore needed to preempt their potential expansion.

### Drug resistance as a factor associated with active transmission

We found that HIV-1 sequences with drug resistance were significantly more likely to be classified as active than non-resistant sequences (*aOR* = 1.777), suggesting that drug-resistant variants may show enhanced persistence and activity within evolving molecular transmission networks. The mechanisms underlying this association remain unclear but may involve both viral and epidemiological factors. Although resistance-associated mutations often impose fitness costs, their effects on *in vivo* transmission vary considerably across mutations. Previous studies using large-scale molecular transmission networks demonstrated that several common resistance mutations, including K103N, Y181C, and L90M, showed transmission fitness comparable to or greater than that of wild-type viruses, enabling the establishment of persistent drug-resistant reservoirs (Wertheim et al., 2017). Therefore, resistant variants with limited fitness impairment may retain sufficient replicative capacity to circulate efficiently and contribute to ongoing transmission.

Additionally, the higher active proportion of drug-resistant sequences may be associated with their distribution within specific transmission sub-populations and subtypes. In China, major circulating subtypes, including CRF07_BC, CRF01_AE, CRF65_cpx, and CRF55_01B, have been shown to form large transmission clusters, some of which contain a high proportion of sequences carrying resistance-associated mutations (Hong et al., 2023). Drug-resistant variants concentrated in highly connected networks may have greater opportunities to establish links with newly reported cases. Thus, the increased active proportion of resistant sequences may reflect the combined effects of viral characteristics, network structure, and host transmission behaviors. These findings support integrating resistance surveillance with longitudinal molecular network analysis, as identifying active resistant sequences may help detect actively spreading resistant variants and guide targeted public health interventions to limit the onward transmission of HIV-1 drug resistance.

### Methodological strengths of machine learning in HIV molecular epidemiology

In this exploratory study, we applied gradient boosting to identify factors associated with active sequences within an HIV-1 molecular transmission network, an approach that offers several methodological advantages pertinent to HIV molecular epidemiology. First, as a tree-based ensemble method, gradient boosting automatically captures complex and nonlinear interactions among demographic, behavioral, and virological factors without requiring pre-specified models or extensive feature engineering. Second, SHAP-based importance analysis identified HIV-1 subtype, age at diagnosis, drug resistance, and sex as the top contributing predictors, providing an interpretable and robust ranking that corroborated the multivariable logistic regression findings. Third, computing SHAP values out-of-fold across the 10 cross-validation folds provided a transparent assessment of model stability and ensured that variable importance estimates were not overfitted to the training data. Together, these methodological features make gradient boosting a valuable analytical tool for HIV molecular epidemiology, particularly for identifying actively transmitting populations and guiding targeted resource allocation.

### Public health implications

The active node definition used in this study resembles the active cluster method, in that both identify potential transmitters through genetic links to newly reported cases. The advantage of the node-level strategy lies in its ability to directly pinpoint individuals directly, thereby avoiding the false positives inherent in cluster-level definitions, which may include inactive nodes. Furthermore, the node-level rule is transparent and reproducible, facilitating standardized implementation across different surveillance settings. This precision-focused approach conserves limited public health resources and is particularly feasible in resource-constrained settings. Thus, the node-level definition serves as a refined and practical complement to cluster-based approaches.

The findings suggest several implications for HIV prevention in Hainan. First, routine screening and adherence support should prioritize older adults (≥50 years), given their high proportion of active sequences and vulnerability to delayed diagnosis. Second, enhanced surveillance should target the less prevalent subtypes, particularly CRF65_cpx and unique recombinant forms, which had the highest proportions of active sequences and merit priority in cluster response. Third, baseline resistance testing should be strengthened for individuals whose sequences are classified as active, as resistant strains are more likely to propagate within clusters. Finally, tailored outreach to sexually active males, including MSM and migrant workers, is recommended, given the marked male predominance in active transmission.

### Limitations

Several limitations of this study should be noted. First, the molecular transmission network is inherently dependent on sampling coverage. Undiagnosed or unsampled individuals are excluded, potentially introducing bias in network metrics. Second, the analytical results are sensitive to the genetic distance threshold used for network construction, and although we selected 0.009 based on cluster count, different thresholds might yield alternative network configurations. Third, because newly reported cases are defined by report date rather than infection date, the active label reflects network connectivity and reporting activity rather than the biological recency of infection. Future studies incorporating recency assays could refine this definition. Fourth, the node-level approach is limited in settings with low sampling coverage, where genuinely transmitting individuals may not be labeled as active because intermediate links remain unsequenced, leading to under-identification of certain cases. This limitation can be partially addressed by improving surveillance coverage in future studies. Finally, due to significant differences between individuals included in the network and those not included in terms of sex, age at diagnosis, and HIV-1 subtype, our findings may not generalize to individuals without sequence data.

## Conclusions

Age ≥50 years, HIV-1 subtype, drug resistance, and male sex were independently associated with active sequences. Our findings support enhanced surveillance of the less prevalent subtypes (CRF65_cpx and Crecombinant forms), baseline drug resistance testing, routine screening for older adults, and targeted outreach to sexually active males. Integration of molecular network data into public health practice and future external validation studies are warranted.

## Data Availability

The original contributions presented in the study are publicly available. This data can be found in GenBank (https://www.ncbi.nlm.nih.gov/genbank/) under the accession numbers. The accession numbers and their corresponding sample identifiers can be found in the article/**Supplementary Material**.
