# Supplementary material for: Identification of active sequences in HIV-1 molecular transmission networks using machine learning: a cross-sectional study in Hainan Province, south China
Source: Front Cell Infect Microbiol. 2026 Oct 1;16:1925404. doi: 10.3389/fcimb.2026.1925404 (PMC13668595; doi:10.3389/fcimb.2026.1925404)
Supplement: Supplementary file 1 [file Table2.docx]

**Supplementary Material**

*Tables S1–S8, numbered in order of first citation in the manuscript*

**Table S1. Primers for HIV-1 *pol* region amplification**

| **Round** | **Name** | **Sequence (5' to 3')** | **HXB2 position** | **Direction** |
| --- | --- | --- | --- | --- |
| First round | F1a | TGAARGA/ideoxyI/TGYACTGARAGRCAGGCTAAT | 2057-2085 | Forward |
|  | F1b | ACTGARAGRCAGGCTAATTTTTTAG | 2068-2092 | Forward |
|  | 21-01 | GTAYTTCYGCYTAAGTCTTTTGATGGG | 3509-3537 | Reverse |
| Second round | O1-01 | CAGAGCCAACAGCCCCACCA | 2147-2166 | Forward |
|  | RT20-01AE | CTGCCAAYTCTAATTCTGCTTC | 3441-3462 | Reverse |

**Table S2. Reaction mixtures for the first- and second-round amplification of the HIV-1 *pol* gene.**

| **Round** | **Whole-blood DNA** | **Plasma RNA** |
| --- | --- | --- |
| First round | Mix | Mix^a^ |
|  | F1a | Buffer^a^ |
|  | F1b | F1a |
|  | 21-01 | F1b |
|  | H_2_O | 21-01 |
|  | DNA | H_2_O |
|  | — | RNA |
| Second round | Mix | |
|  | O1-01 | |
|  | RT20-01AE | |
|  | The first round products | |
|  | H_2_O | |

The first round of PCR was performed with genomic DNA, while the first round of RT-PCR was performed with RNA. The second round of PCR used the first-round PCR products as template. ^a^PrimeScript One Step RT‑PCR Kit Ver.2 was purchased from TaKaRa Bio Inc. (Dalian, China). “—” indicates the component was not added. All primers were synthesized by Sangon Biotech (Shanghai) Co., Ltd.

**Table S3. Thermal cycling protocols for HIV-1 *pol* gene amplification.**

| **Thermal cycling protocol** | **DNA nested PCR** | **RNA RT-PCR** | **Cyclic number** |
| --- | --- | --- | --- |
| First round | 94℃, 5min | 50℃, 30min | 1× |
|  | — | 94℃, 3min |  |
|  | 94℃, 30sec | 94℃, 30sec | 35× |
|  | 50℃, 30sec | 50℃, 30sec |  |
|  | 72℃, 2min | 72℃, 2min |  |
|  | 72℃, 10min | 72℃, 10min | 1× |
|  | 8℃, ∞ | 4℃, ∞ |  |
| Second round | 94℃, 5min | 94℃, 3min | 1× |
|  | 94℃, 30sec | 94℃, 30sec | 32× |
|  | 63℃/55℃, 30sec | 63℃, 30sec |  |
|  | 72℃, 2.5min | 72℃, 2.5min |  |
|  | 72℃, 10min | 72℃, 10min | 1× |
|  | 8℃, ∞ | 4℃, ∞ | — |

First-round and second-round thermal cycling conditions for nested PCR (DNA template) and RT-PCR (RNA template) are shown side by side. The cyclic number column indicates the number of cycles for each step. “×” denotes the number of cycles performed. “—” indicates that no corresponding step was performed; “∞” indicates an indefinite hold at the indicated temperature. Detailed primer sequences and reaction mixture compositions are provided in Tables S1 and S2, respectively.
